# Identification of a novel mutation in the SERPING1 gene in a 17-year-old Chinese girl with type I hereditary angioedema

**DOI:** 10.1016/j.jdcr.2024.10.023

**Published:** 2024-11-10

**Authors:** Wei Zhang, Hongwei Liu

**Affiliations:** Department of Dermatology, Henan Provincial People′s Hospital, Zhengzhou University People′s Hospital, Henan University People′s Hospital, Zhengzhou, China

**Keywords:** Chinese, hereditary angioedema, mutation, SERPING 1 gene

## Introduction

Hereditary angioedema (HAE) is a rare autosomal dominant genetic disease, which is characterized by recurrent unpredictable and potentially life-threatening subcutaneous edema, usually involving skin, gastrointestinal tract, or airway tissues. Literature reports that the prevalence rate of HAE is about 1.5/100,000,^1^ and there is still lack of epidemiologic data in China. HAE can be divided into 2 categories: HAE-C1-INH (HAE due to C1 inhibitor deficiency) and HAE-nC1-INH (HAE with normal C1 inhibitor). HAE-C1-INH can be divided into type 1 HAE, which has a reduced concentration of C1-INH and low function, and type 2 HAE, which has an impaired C1-INH function despite a normal or even elevated concentration of C1-INH. Mutations of SERPING1 gene encoding C1-INH is the cause of most cases of HAE. We report here a novel SERPING 1 gene mutation that causes type 1 HAE.

**Fig 1:**
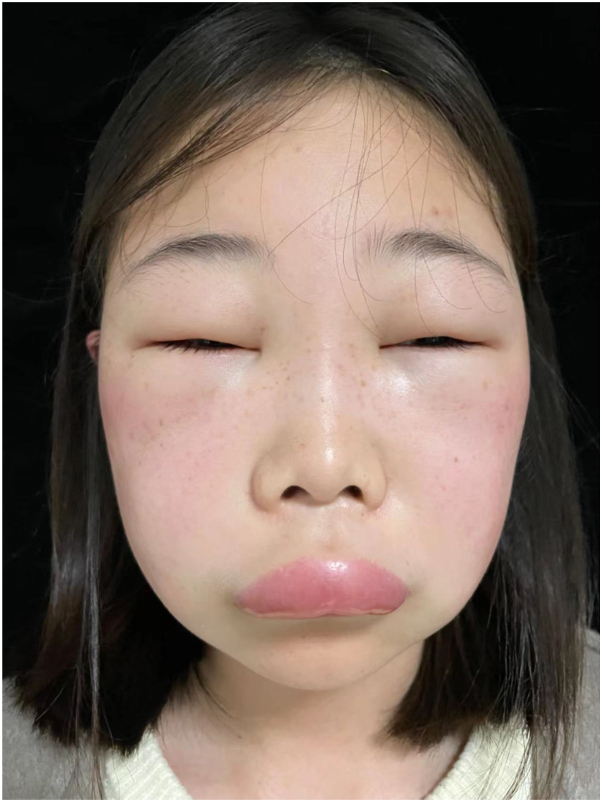
Clinical image. Flushed and swollen face, accompanied by edema of both eyelids and lips.

**Fig 2:**
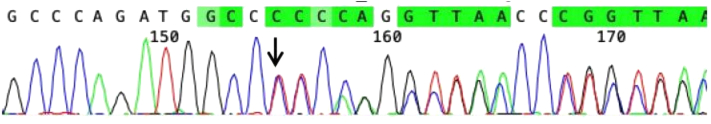
Sequencing map. Heterozygous frameshift mutation of SERPING1 gene (NM_000062.3) c.6dup in peripheral blood samples of patients.

This is a 17-year-old girl who complained of recurrent intermittent facial edema and dyspnea for several years without allergic history, and the symptoms cannot be alleviated after using antihistamines or dexamethasone. Physical examination (Fig 1) revealed facial edema with erythema, including edema of both eyelids and lips. Additionally, she had suffered from abdominal pain several times since the age of 8. Laboratory findings showed that complete blood count, c-reactive protein, erythrocyte sedimentation rate, antinuclear antibody titer + extractible nuclear antigen test, serum immunoglobulin (Ig)A, IgG, IgM, C3, total IgE, and specific IgE (The specific IgE names are as follows: protein, milk, peanuts, fish, wheat, soybeans, cat dander, dog dander, horse dander, cow dander, house dust, dust mites, dust mites, German cockroaches, Aspergillus flavus, Aspergillus fumigatus, Alternaria alternata, Candida albicans, peanuts, hazelnuts, Brazil nuts, almonds, coconuts, grey alders, European hazelnuts, American elms, willows, American boxwoods, common ragweed, mugwort, French chrysanthemum, dandelion.) were normal, but C4 was as low as 0.03 g/L (normal range: 0.1-0.4 g/L). Serum IL-5 was 3.59pg/ml (normal range:≤3.1pg/ml). Additionally, the test using dry blood stains showed a complement C4 concentration of 19.37ug/ml (normal range: 72.85-372.95ug/ml), a C1 inhibitor function of<7% (normal range: ≥ 58.9), and a C1 inhibitor concentration of 25.78ug/ml (standard range: 81.46-291.29ug/ml), supporting a diagnosis of type 1 HAE. The function and concentration of C1 inhibitor and complement C4 concentration of the patient's parents and younger brother were normal. Based on the fact that immediate and closest relatives within 3 generations did not have the same disease history, we considered the possibility of a genetic mutation during pregnancy. To further evaluate, gene sequence analysis was carried out according to the Standardized Operating Procedures for Genetic Diagnosis of Genetic Diseases. Genomic DNA of peripheral blood samples was extracted, and the coding region of SERPING1 gene (NM_000,062.3) was detected by PCR amplification and Sanger sequencing. A heterozygous frameshift mutation NM_000,062.3: c.6dup p. Ser3Leufs∗17 in SERPING1 gene at chr11:57,365,749 was identified (Fig 2). Based on the Human Gene Mutation Database, the Leiden Open Variation Database LOVD, GeneCards and henan province prenatal diagnosis center, this novel variant in the SERPING1 gene was identified. More than 1000 SERPING1 gene variants related to HAE have been identified. However, only about 60 variants were found in Chinese population.^2^^,^^3^ The incidence of laryngeal edema in HAE patients in China is as high as 59%.^4^ The average time from edema to asphyxia is 4.6 h, with a fatality rate of 92.2%.^5^ Considering that this patient experienced facial edema 2-3 times per month, most of which were accompanied by dyspnea, the patient was given prophylactic treatment with subcutaneous injection of lanadelumab and had no recurrence during the current 4-month follow-up period (Supplementary Material, available via Mendeley at https://data.mendeley.com/datasets/8r3zvmjzhf/1).

## Conflicts of interest

None disclosed.
